# Kuru likened to scrapie: the story remembered

**DOI:** 10.1098/rstb.2008.4013

**Published:** 2008-11-27

**Authors:** William J. Hadlow

**Affiliations:** 908 South Third StreetHamilton, MT 59840-2924, USA

In my letter to the editor of the *Lancet* of 5 September 1959, I pointed out the striking similarity of kuru and scrapie and suggested that kuru, like scrapie, might be a transmissible disease expressed after a long incubation period (Hadlow 1959). The unlikely linkage of these two diseases came about fortuitously while I was an employee of the United States Department of Agriculture studying the pathology of scrapie at the then Agricultural Research Council Field Station at Compton in Berkshire, England, beginning in March 1958.

A year later parasitologist William Jellison, a friend and colleague from Rocky Mountain Laboratory, Hamilton, Montana, where I had worked before coming to England, visited my wife and me in Compton on the afternoon of 28 June 1959. He was returning from meetings in Eastern Europe. At dinner that evening he casually mentioned an exhibit he saw the previous day at the Wellcome Medical Museum in London. It had to do with a strange brain disease affecting the primitive people in Papua New Guinea. He thought I might like to see it owing to my interest in neuropathology.

Five days later I saw the exhibit in London. It was on kuru, the strange brain disease, describing its clinical features and illustrating the neuropathological changes in its victims. Neuronal degeneration and intense astrocytosis likened kuru to scrapie. The likeness was made even more so by the single and multilocular vacuoles in the perikaryon of large neurons. From the start I was drawn to them, for they were so much like those in scrapie (Hadlow 1992).

Although unusual in human neuropathology, in veterinary pathology such vacuolated neuronal cell bodies had long been identified almost solely with scrapie. Indeed, they had become the well-entrenched neurohistological hallmark of the disease and were (and still are) counted on in making its post-mortem diagnosis. Because spongiform change in the grey matter neuropil lacked prominence in both kuru and scrapie at the time, it was not part of the resemblance I saw in them. Then too, the topographic pattern of the degenerative changes in the brain has a certain sameness in the two diseases, with accent on the cerebellar cortex. So whatever else kuru and scrapie have in common—similar natural histories and general clinical features—the essential link between them was neurohistological, as tenuous as that seemed then and later (Hadlow 1995).

The close kinship of kuru and scrapie, only surmised in 1959, was made more certain when the experimental transmission of kuru to chimpanzees was reported in 1966. It was indeed a welcome revelation. As it turned out kuru and scrapie are broadly similar transmissible neurological diseases.

Two years earlier scrapie gave up its long-held exclusiveness as the only known transmissible degenerative disease of the central nervous system when a rare neurological disease of ranch mink was shown to be cast in the scrapie mould. But here was a human disease cast in that mould—the first one. The distinction had unforeseen implications, not least in helping bring together seemingly diverse neurological diseases of man and animals now commonly called the transmissible spongiform encephalopathies (Hadlow 1999).

That my letter in the *Lancet* had a role, however small it may have been, in identifying other diseases belonging to this distinctive family, of course, pleases me. After all it was but the consequence of a chance observation. Still, knowing something about scrapie, especially its pathology, helped me to see the resemblance of the two diseases at first glance. I am sure that would not have happened as readily had I not learned a lot about scrapie in sheep and goats and looked at many of their brains microscopically during the previous year, for I came to Compton knowing little about the disease. Nevertheless, the overall likeness of kuru and scrapie is such that no doubt sooner or later someone would have become aware of it, though probably not as I did.

These sentiments highlight the story of the kuru–scrapie likeness as I remember it.
